# Supplementary material for: Dynamic early recruitment of GAK–Hsc70 regulates coated pit maturation
Source: Proc Natl Acad Sci U S A. 2025 May 27;122(22):e2503738122. doi: 10.1073/pnas.2503738122 (PMC12146748; doi:10.1073/pnas.2503738122)
Supplement: Supplementary file 1 [file pnas.2503738122.sapp.pdf]

SI Appendix for:

Dynamic early recruitment of GAK-Hsc70 regulates coated pit maturation

Zhangping He, Peiyao Zuo, Peiliu Xu, Haozhi Yuan, Madhura Bhave, Xiangying Wei, Ziyan Yang, Lu Han, Sandra L. Schmid*, Zhiming Chen*

*To whom correspondence may be addressed.

Email: sandra.schmid@czbiohub.org; Zhiming.Chen@usc.edu.cn

Supplemental Movies

Movie 1: Time-lapse TIR-FM imaging of ARPE-HPV eGFP-CLCa cells treated with control siRNA (left) or GAK siRNA (middle), as well as ARPE-HPV eGFP-CLCa+GAK(WT)-mRuby cells that were treated with GAK siRNA (right). Images were obtained at 1 frame/s and collected for 7.5 min. Movie is accelerated 25-fold.

Movie 2: Time-lapse TIR-FM imaging of ARPE-HPV eGFP-CLCa+GAK(WT)-mRuby cells (#1 from left), ARPE-HPV eGFP-CLCa+GAK(Kinase*)-mRuby cells (#2 from left), ARPE-HPV eGFP-CLCa+GAK(PTEN*)-mRuby cells (#3 from left) and ARPE-HPV eGFP-CLCa+GAK(AP2*)-mRuby cells (#4 from left) that were treated with GAK siRNA. Images were obtained at 1 frame/s and collected for 7.5 min. Movie is accelerated 25-fold.

Movie 3: Time-lapse TIR-FM imaging of ARPE-HPV eGFP-CLCa+GAK(WT)-mRuby cells (left), ARPE-HPV eGFP-CLCa+GAK(clathrin*)-mRuby cells (middle) and ARPE-HPV eGFP-CLCa+GAK(J*)-mRuby cells (right) that were treated with GAK siRNA. Images were obtained at 1 frame/s and collected for 7.5 min. Movie is accelerated 25-fold.

Supplemental Figures:

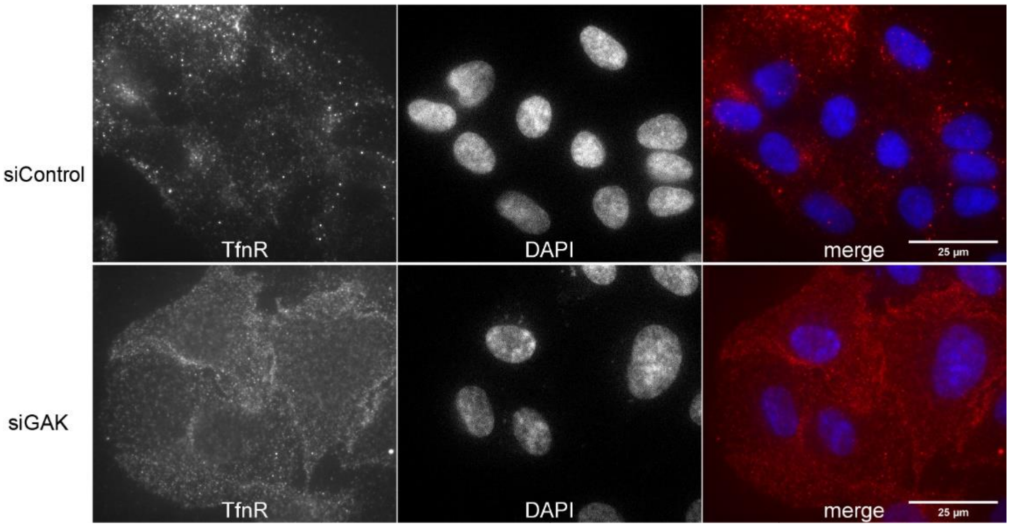

Figure S1. GAK knockdown leads to the accumulation of TfnR on the surface of ARPE-HPV eGFP-CLCa cells. Images shown were acquired by immunofluorescence and TIRFM imaging. Scale bars = 25 µm.

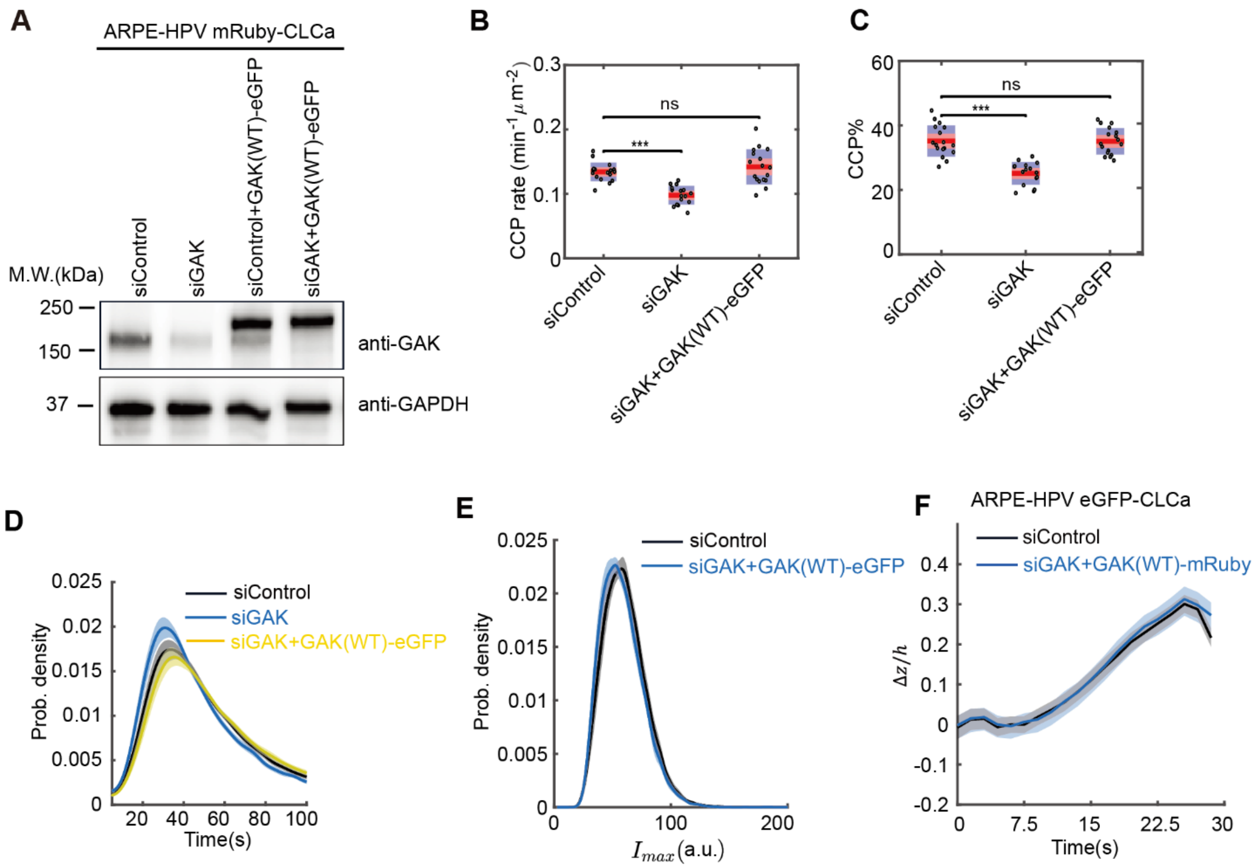

Figure S2. Expression of GAK-eGFP(WT) or GAK-mRuby(WT) rescues the phenotypes of GAK knockdown. (A) Western blot indicates the expression level of exogenous GAK-eGFP in ARPE-HPV mRuby-CLCa cells and the efficiency of 3' UTR siRNA-mediated knockdown of GAK. **(B-C)** Expression of GAK(WT)-eGFP rescued the reduction in the initiation rate and % of CCPs induced by GAK knockdown. Statistical analysis of the data in (B) and (C) is the Wilcoxon rank sum test, ns: P > 0.05; ***: P<0.001. **(D-F)** Expression of GAK(WT)-eGFP restored (D) the lifetime distribution, (E) the maximum fluorescence intensity distribution, and (F) the invagination depth of *bona fide* CCPs to control values. Data presented were obtained from n = 15 movies for each condition. Each dot in (B,C) represents a movie. Number of dynamic tracks analyzed IN (B-E): 180838 for siControl, 185121 for siGAK, 187323 for siGAK+GAK(WT)-eGFP. Number of CCP tracks analyzed to obtain the $\Delta z/h$ curves in (F): 15,395 for siControl, 10,880 for siGAK+GAK(WT)-mRuby. Shadowed area in (D-F) indicates 95% confidential interval.

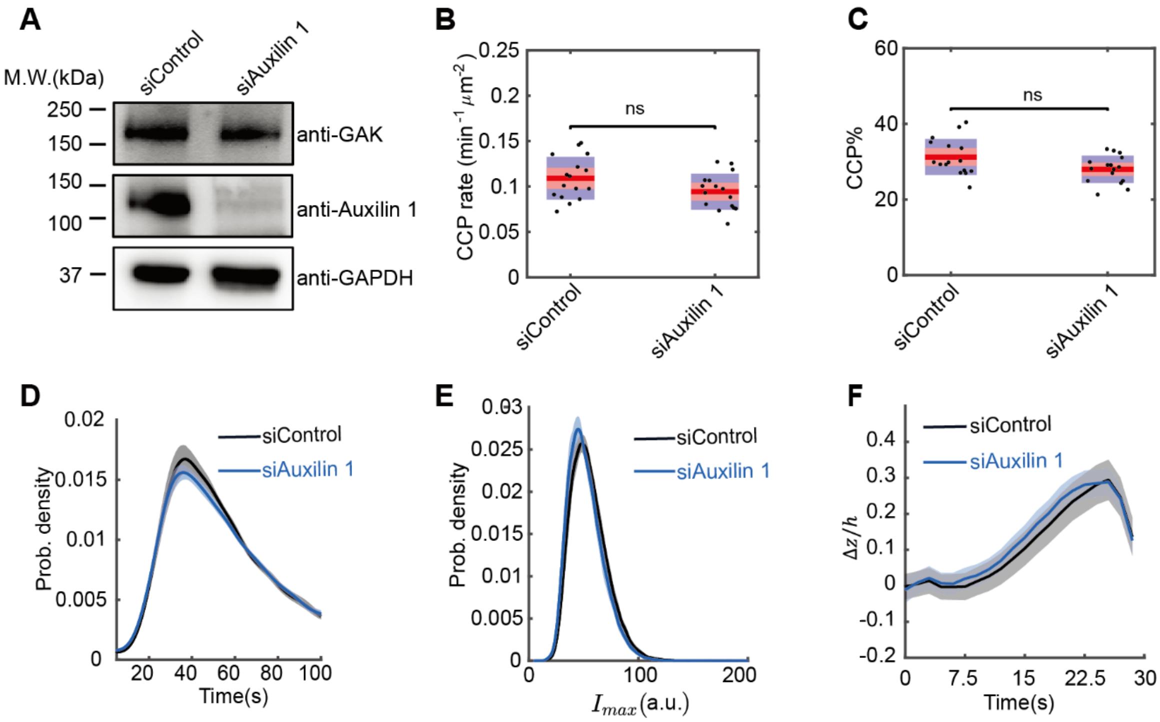

Figure S3. Auxilin 1 Knockdown does not affect CCP formation or invagination. (A) siRNA-mediated knockdown efficiency of Auxilin 1 in ARPE-HPV eGFP-CLCa cells probed by western blotting. Auxilin 1 knockdown did not affect GAK levels. **(B-C)** Auxilin 1 knockdown did not affect the initiation rate and % of bona fide CCPs. Statistical analysis of the data in (B) and (C) is the Wilcoxon rank sum test, ns: P > 0.05. **(D-F)** Auxilin 1 knockdown did not affect (D) the lifetime distribution, (E) the maximum fluorescence intensity distribution, or (F) the invagination depth of *bona fide* CCPs. Data presented were obtained from n = 15 movies for each condition. Each dot represents a movie. Number of dynamic tracks analyzed:166,745 for siControl, 149,623 for siAuxilin 1. Number of CCP tracks analyzed to obtain the $\Delta z/h$ curves in (F): 16,972 for siControl, 8,332 for siAuxilin 1. Shadowed area in (D-F) indicates 95% confidential interval.

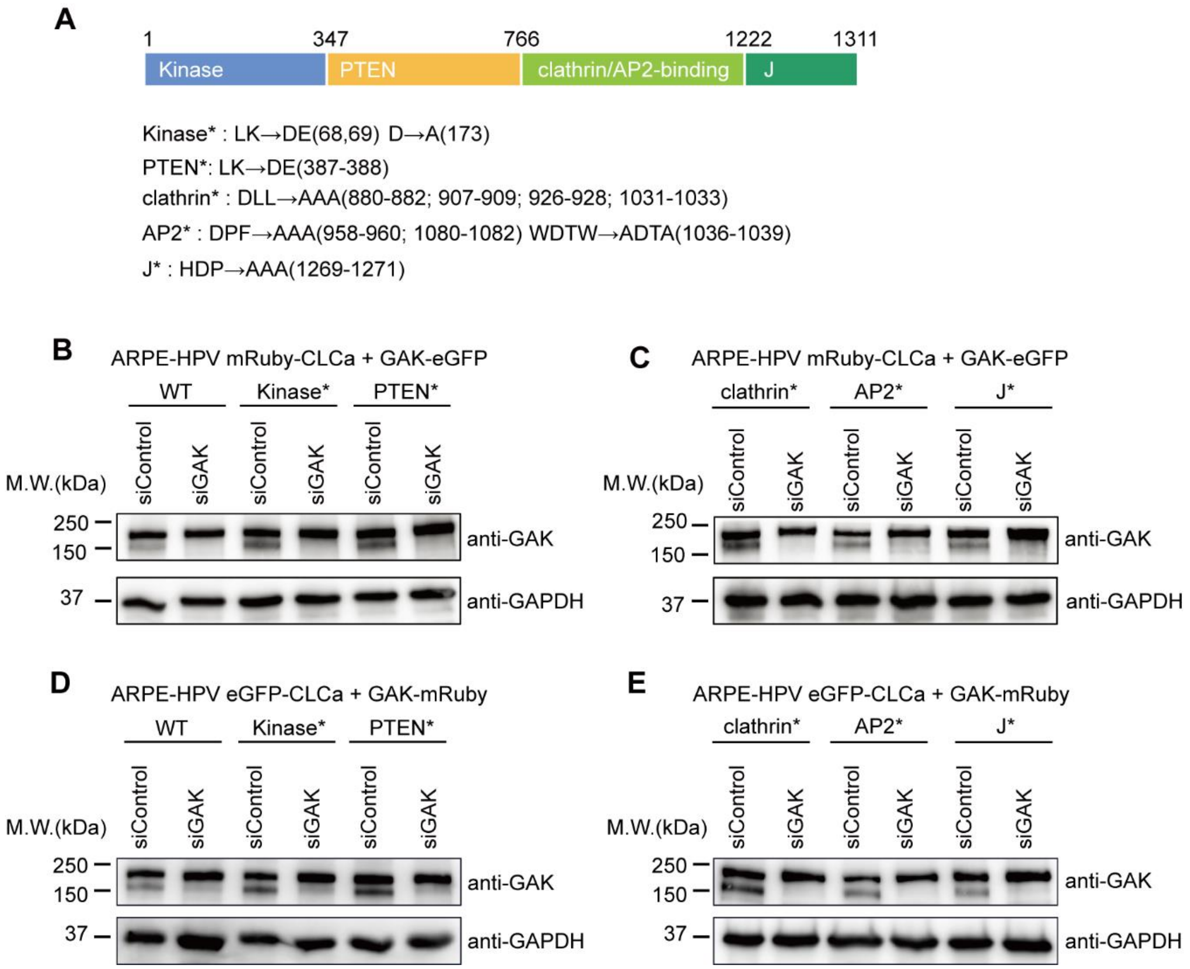

Figure S4. Generation of GAK mutants and stable cell lines. (A) Domain structure of GAK and corresponding mutations (denoted with *) to abolish domain functions. **(B-C)** Western blotting of ARPE-HPV mRuby-CLCa cells that stably express GAK-eGFP (WT and mutant constructs) and treated with control siRNA or GAK siRNA. Treatment of GAK siRNA knocked down endogenous but not exogenous GAK. **(D-E)** Western blotting of ARPE-HPV eGFP-CLCa cells that stably express GAK-mRuby (WT and mutant constructs) and treated with control siRNA or GAK siRNA. Treatment of GAK siRNA knocked down endogenous but not exogenous GAK.

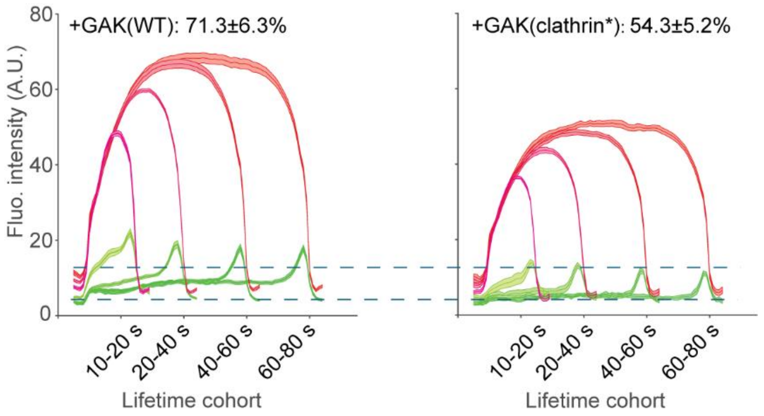

Figure S5. Recruitment of GAK(clathrin*) to CCPs is inhibited but not abolished when compared with GAK(WT). Fluorescence intensity cohorts of mRuby-CLCa (red) and GAK-eGFP (green) were generated using dual-channel TIRFM imaging and cmeAnalysis. GAK(WT) was recruited to 71.3±6.3% CCPs while GAK(clathrin*) was recruited to 54.3±5.2% CCPs. In addition, the magnitude of GAK(WT) recruitment was higher than that of GAK(clathrin*).

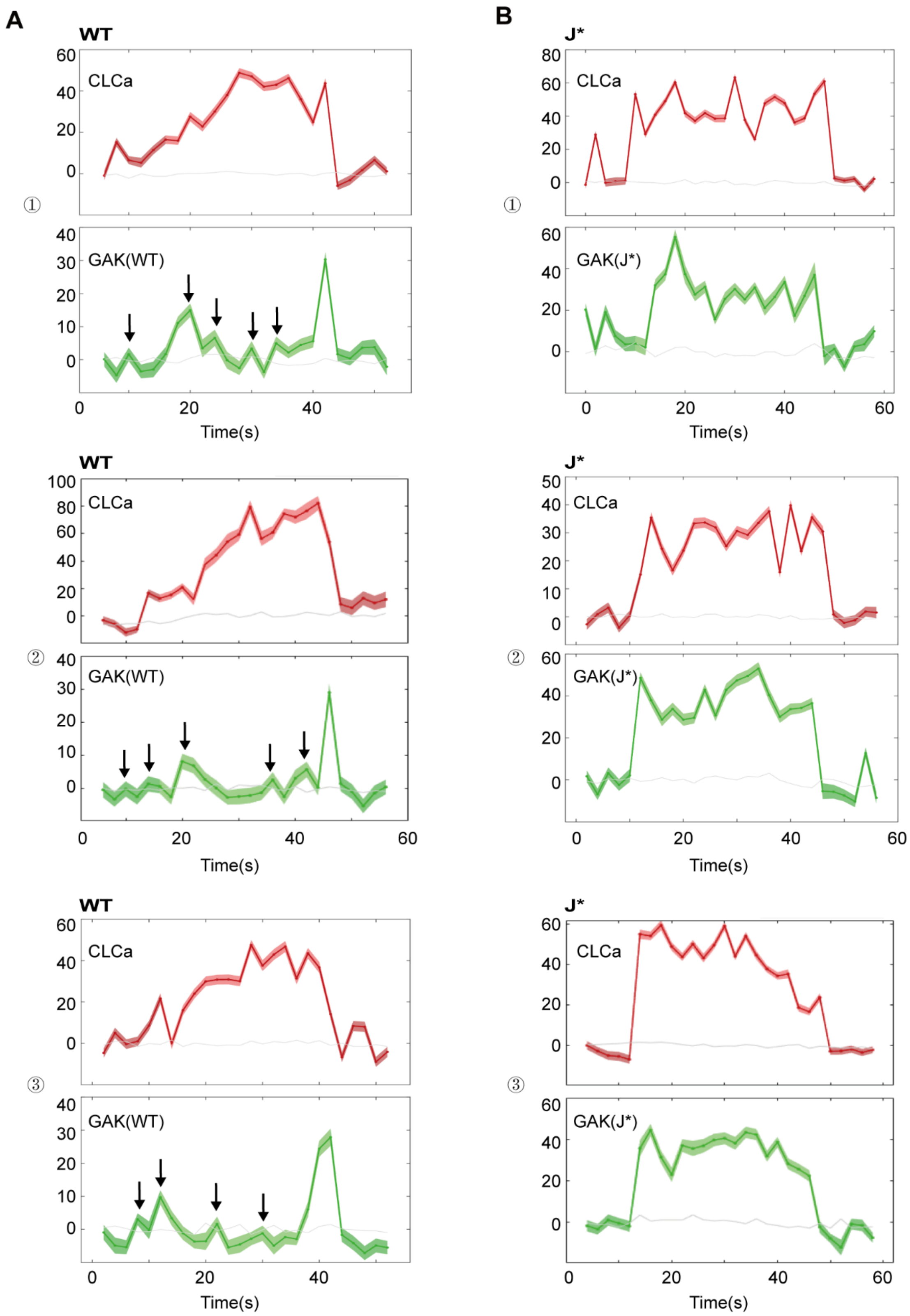

Figure S6. Additional representative dual-channel (mRuby-CLCa and eGFP-GAK) tracks of CCPs from ARPE-HPV mRuby-CLCa cells that stably express GAK-eGFP: **(A)** WT and **(B)** J*.

**Mutagenesis**	**Forward primer**	**Reverse primer**
Kinase：LK(68,69)→DE*	*AGATCTGGGAAGTGGCAGAGAGTATGCAGACGAGCGATTACTATCCAATGAAG*	*CTTCATTGGATAGTAATCGCTCGTCTGCATACTCTCTGCCACTTCCCAGATCT*
Kinase：D(173)→A*	*CACCCATCATCCACAGAGCTCTCAAGGTTGAAAACTT*	*AAGTTTTCAACCTTGAGAGCTCTGTGGATGATGGGTG*
PTEN：LK (387,388)→DE*	*GAACGGCTCTTCACCAACGACGAGGATACTTCCTCCAAGG*	*CCTTGGAGGAAGTATCCTCGTCGTTGGTGAAGAGCCGTTC*
clathrin：DLL (880-882)→AAA*	AGCCCGAAGAAGGTATTGXTACCGCGGGGCTACACTCTGAGGG	CCCTCAGAGTGTAGCCCCGCGGCAGCAACACCTTCTTCGGGCT
clathrin：DLL (907-909)→AAA*	GGGTCCCATCTAGCAACACTGCCGCGGCGAGCTGCCTTCTTGAACCATC	GATGGTTCAAGAAGGCAGCTCGCCGCGGCAGTGTTGCTAGATGGGACCC
clathrin：DLL (926-928) →AAA*	AGTAGGGCCTCCTGGTGCCGCGGCTGGTGGTGAGGCTCCA	GTGGAGCCTCACCACCAGCCGCGGCACCAGGAGGCCCTACT
clathrin：DLL (1031-1033) →AAA*	GATTCATCCAGCCACCCAGCTGCGGCAGGAGGATGGGATACGTGG	CCACGTATCCCATCCTCCTGCCGCAGCTGGGTGGCTGGATGAAGC
AP2：DPF (958-960) →AAA*	GAAAAGTCCCTGACACTGTGGCCGCAGCAGACCAGTTCCTGCTGTCATCC	GGATGACAGCAGGAACTGGTCTGCTGCGGCCACAGTGTCAGGGACTTTTC
AP2：DPF (1080-1082) →AAA*	CCAAACCAAGTCTCAGAACCTTGCCGCAGCAGCTGATCTCAGTGATCTCAGCTC	GAGCTGAGATCACTGAGATCAGCTGCTGCGGCAAGGTTCTGAGACTTGGTTTGG
AP2：WDTW (1036-1039) →ADTA*	CCAGATCTGCTAGGAGGAGCGGATACGGCGGCTGACACAGCTAC	GTAGCTGTGTCAGCCGCCGTATCCGCTCCTCCTAGCAGATCTGG
J：HPD (1269-1271) →AAA*	GTGCAGTGCTGGTAGTGGCTGCTGCTAAGGCCACAGGGCAGC	GCTGCCCTGTGGCCTTAGCAGCAGCCACTACCAGCACTGCAC
**Cloning**	**Forward primer**	**Reverse primer**
backbone：pLVx-IRES-Puro	*GAGGATCTATTTCCGGTGCCACCATGTCGCTGCTGCAGTC*	*CCTCGCCCTTGCTCACCATACTACTTCCACTACCAGTTGATCC*
insert gene：GAK(WT)		

insert gene : eGFP	*ACTGGTAGTGGAAGTAGTATG GTGAGCAAGGGCGA*	*GGCCGCTCTAGAACTAGTTTACTT GTACAGCTCGTCCATGC*
backbone : pLVx-IRES-Puro-RFP670	*GAGGATCTATTTCCGGTGCCA CCATGTCGCTGCTGCAGTC*	*CCTCGCCCTTGCTCACCATACTAC TTCCACTACCAGTTGATCC*
insert gene : GAK(Kinase)*		
insert gene : eGFP	*ACTGGTAGTGGAAGTAGTATG GTGAGCAAGGGCGA*	*GGCCGCTCTAGAACTAGTTTACTT GTACAGCTCGTCCATGC*
backbone : pLVx-IRES-Puro	*GAGGATCTATTTCCGGTGCCA CCATGTCGCTGCTGCAGTC*	*CCTCGCCCTTGCTCACCATACTAC TTCCACTACCAGTTGATCC*
insert gene : GAK(PTEN)*		
insert gene : eGFP	*ACTGGTAGTGGAAGTAGTATG GTGAGCAAGGGCGA*	*GGCCGCTCTAGAACTAGTTTACTT GTACAGCTCGTCCATGC*
backbone : pLVx-IRES-Puro	*GAGGATCTATTTCCGGTGCCA CCATGTCGCTGCTGCAGTC*	*CCTCGCCCTTGCTCACCATACTAC TTCCACTACCAGTTGATCC*
insert gene : GAK(Clathrin)*		
insert gene : eGFP	*ACTGGTAGTGGAAGTAGTATG GTGAGCAAGGGCGA*	*GGCCGCTCTAGAACTAGTTTACTT GTACAGCTCGTCCATGC*
backbone : pLVx-IRES-Puro	*GAGGATCTATTTCCGGTGCCA CCATGTCGCTGCTGCAGTC*	*CCTCGCCCTTGCTCACCATACTAC TTCCACTACCAGTTGATCC*
insert gene : GAK(AP2)*		
insert gene : eGFP	*ACTGGTAGTGGAAGTAGTATG GTGAGCAAGGGCGA*	*GGCCGCTCTAGAACTAGTTTACTT GTACAGCTCGTCCATGC*
backbone : pLVx-IRES-Puro	*GAGGATCTATTTCCGGTGCCA CCATGTCGCTGCTGCAGTC*	*CCTCGCCCTTGCTCACCATACTAC TTCCACTACCAGTTGATCC*
insert gene : GAK(J)*		
insert gene : eGFP	*ACTGGTAGTGGAAGTAGTATG GTGAGCAAGGGCGA*	*GGCCGCTCTAGAACTAGTTTACTT GTACAGCTCGTCCATGC*
**Cloning**	**Forward primer**	**Reverse primer**
backbone : pLVx-IRES-Puro-RFP670	*CAACTGGTAGTGGAAGTAGTA TGGTGTCTAAGGGCGAAGAG*	*GCGGCCGCTCTAGAACTAGTCTA CTTGTACAGCTCGTCCATCCC*
insert gene : mRuby		
insert gene : GAK(WT)	*CTAGAGGATCTATTTCCGGTG CCACCATGTCGCTGC*	*TCTTCGCCCTTAGACACCATACTA CTTCCACTACCAGTTGATCCTG*
backbone : pLVx-IRES-Puro-RFP670	*CAACTGGTAGTGGAAGTAGTA TGGTGTCTAAGGGCGAAGAG*	*GCGGCCGCTCTAGAACTAGTCTA CTTGTACAGCTCGTCCATCCC*

insert gene : mRuby		
insert gene : GAK(Kinase)*	*CTAGAGGATCTATTTCCGGTGCCACCATGTCGCTGC*	*TCTTCGCCCTTAGACACCATACTACTTCCACTACCAGTTGATCCTG*
backbone : pLVx-IRES-Puro-RFP670	*CAACTGGTAGTGGAAGTAGTATGGTGTCTAAGGGCGAAGAG*	*GCGGCCGCTCTAGAACTAGTCTACTTGTACAGCTCGTCCATCCC*
insert gene : mRuby		
insert gene : GAK(PTEN)*	*CTAGAGGATCTATTTCCGGTGCCACCATGTCGCTGC*	*TCTTCGCCCTTAGACACCATACTACTTCCACTACCAGTTGATCCTG*
backbone : pLVx-IRES-Puro-RFP670	*CAACTGGTAGTGGAAGTAGTATGGTGTCTAAGGGCGAAGAG*	*GCGGCCGCTCTAGAACTAGTCTACTTGTACAGCTCGTCCATCCC*
insert gene : mRuby		
insert gene : GAK(Clathrin)*	*CTAGAGGATCTATTTCCGGTGCCACCATGTCGCTGC*	*TCTTCGCCCTTAGACACCATACTACTTCCACTACCAGTTGATCCTG*
backbone : pLVx-IRES-Puro-RFP670	*CAACTGGTAGTGGAAGTAGTATGGTGTCTAAGGGCGAAGAG*	*GCGGCCGCTCTAGAACTAGTCTACTTGTACAGCTCGTCCATCCC*
insert gene : mRuby		
insert gene : GAK(AP2)*	*CTAGAGGATCTATTTCCGGTGCCACCATGTCGCTGC*	*TCTTCGCCCTTAGACACCATACTACTTCCACTACCAGTTGATCCTG*
backbone : pLVx-IRES-Puro-RFP670	*CAACTGGTAGTGGAAGTAGTATGGTGTCTAAGGGCGAAGAG*	*GCGGCCGCTCTAGAACTAGTCTACTTGTACAGCTCGTCCATCCC*
insert gene : mRuby		
insert gene : GAK(J)*	*CTAGAGGATCTATTTCCGGTGCCACCATGTCGCTGC*	*TCTTCGCCCTTAGACACCATACTACTTCCACTACCAGTTGATCCTG*

Table S1: List of primers.
